# Lack of desensitization of the cough reflex in ovalbumin-sensitized rabbits during exercise

**DOI:** 10.1371/journal.pone.0171862

**Published:** 2017-02-09

**Authors:** Angelica Tiotiu, Bruno Chenuel, Laurent Foucaud, Bruno Demoulin, Silvia Demoulin-Alexikova, Christo Christov, Mathias Poussel

**Affiliations:** 1 EA 3450 DevAH - Development, Adaptation and Disadvantage, Cardiorespiratory regulations and motor control, Université de Lorraine, Vandoeuvre-les-Nancy, France; 2 Pulmonology Department, CHRU Nancy, Nancy, France; 3 Pulmonary Function Testing and Exercise Physiology, CHRU Nancy, Nancy, France; 4 Department of Histology, Université de Lorraine, Vandoeuvre-les-Nancy, France; Centre National de la Recherche Scientifique, FRANCE

## Abstract

**Introduction:**

Cough is a major symptom of asthma frequently experienced during exercise but little is known about interactions between cough and exercise. The goal of our study was to clarify the potential modulation of the cough reflex (CR) by exercise in a spontaneously breathing anaesthetized animal model of airway eosinophilic inflammation.

**Materials & methods:**

Ten ovalbumin (OVA) sensitized adult rabbits and 8 controls were studied. The ventilatory response to direct tracheal stimulation, performed both at rest and during exercise was determined to quantify the incidence and the sensitivity of the CR.

Broncho-alveolar lavages (BAL) and cell counts were performed to assess the level of the airway inflammation following OVA-induced sensitization. Exercise was mimicked by Electrically induced hindlimb Muscular Contractions (EMC).

**Results:**

Among 494 tracheal stimulations, 261 were performed at rest and 233 at exercise. OVA challenges in sensitized rabbits caused a significant increase in the percentage of eosinophils (*p* = 0.008) in BAL. EMC increased minute ventilation by 36% and 35% in OVA and control rabbits respectively, compared to rest values. The sensitivity of the CR decreased during exercise compared to baseline in control rabbits (p = 0.0313) while it remained unchanged in OVA rabbits.

**Conclusion:**

The desensitization of the CR during exercise in control rabbits was abolished in OVA rabbits. The precise role of airway inflammation in this lack of CR desensitization needs to be further investigated but it might contribute to the exercise-induced cough in asthmatics.

## Introduction

Cough is a major airway defensive reflex [1, 2]. It is one of the most common medical afflictions for which patients seek medical attention and which remains an unmet need given the lack of effective and safe treatments [3, 4]. Little is known about the modulation of cough associated with human activities, particularly during exercise [5]. The major cardiorespiratory adjustments occurring during exercise [6] undoubtedly may influence the cough reflex (CR). Some evidence suggest that cough is decreased during exercise in healthy subjects [7] as well as in animals models [8]. In contrast, exercise-induced cough is frequently reported by asthmatic patients [9] or even by athletes exhibiting airway inflammation [10], suggesting the potential of airway inflammation to enhance the CR during exercise. As only limited (and sometimes contradictory) information is available concerning the impact of exercise on cough, our study was conceived to develop an animal model as close as possible to the “asthmatic” airway inflammation and to assess the specific role of exercise on the occurrence of stimulated CR. Electrically induced muscle contractions (EMC) have been extensively used in the study of the control of breathing at exercise (animals and human studies). Consequently, it has long been demonstrated that EMC provides a hyperpnea that follows the same characteristics as the physiological increase in ventilation induced by volitional exercise, particularly matched to metabolism. Therefore, the EMC model appears as a valid model of exercise. Our main goal was then to determine whether airway inflammation could modulate the cough-induced by mechanical stimulation of the trachea during experimental exercise in ovalbumin (OVA)-sensitized rabbits compared to controls. We hypothesized that cough may be either increased or at least not decreased during exercise in OVA rabbits while cough is decreased during exercise in control rabbits.

## Materials and methods

All animal procedures (housing and experiments) were approved by the Lorraine University Committee for the Use and Care of Laboratory animals and performed according to Council Directive 86–609 EEC, issued by the Council of the European Communities, under license from “Ministère de l’Agriculture et de la Pêche” and the “Ministère de l’Enseignement Supérieur et de la Recherche” (A5418-03409), and supervision by the “Services Vétérinaires Départementaux de Meurthe et Moselle”. Eighteen anesthetized, tracheotomized and spontaneously breathing New Zealand adult rabbits (weight: 3.07± 0.30 kg; age: 3 months) were studied (SARL HYCOLE—Route de Villers Plouich, 59159 MARCOING, France, http://www.hycole.com/). Among them, 10 were sensitized to ovalbumin (OVA) and 8 were sham sensitized (controls).

### Ovalbumin sensitization protocol and control group

**Fig 1:**
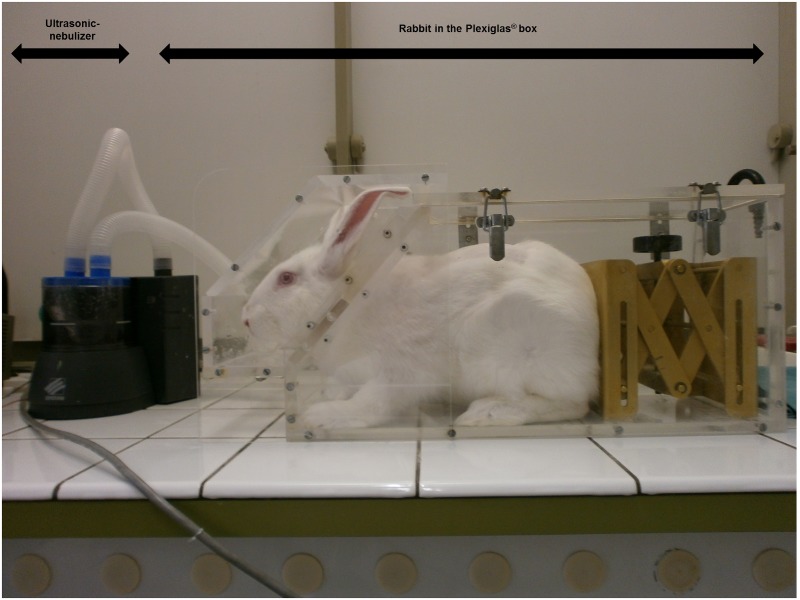
Rabbit exposed to an aerosol administrated in a closed Plexiglas box using an ultrasonic-nebulizer (SYST’AM, LS290). The box was conceived to allow the head of the rabbit to be maintained in front of the aerosol particles and drops cloud generated by the nebulizer. Aerosol sessions were performed 15–20 min daily from day 26 to day 28 either with ovalbumin (OVA rabbits) or with saline (control rabbits).

The month before the acute experiment with tracheal stimulations and exercise, an OVA sensitization protocol was performed. As previously described, OVA sensitized rabbits [11, 12] received intra-peritoneal (i.p.) injections of 0.1 mg OVA and 10 mg Al(OH)_3_ dissolved in 1 ml saline (0.9%) on day 1 and day 14. From day 26 to 28, sensitized rabbits were exposed daily to an aerosol of OVA during 20 minutes (2.5 mg/ml; 50 mg OVA dissolved in 20 ml saline). Aerosols were administrated in a closed Plexiglas box (Fig 1) using an ultrasonic-nebulizer (SYST’AM, LS290). A sham protocol was used on control rabbits. They were treated similarly but saline was systematically used instead of OVA solutions, either intra-peritoneally or in nebulizations. All acute experiments were performed on day 28.

### Ovalbumin sensitization evaluation

#### Intradermal skin tests

Seven days before acute experiments (day 21), sensitization to OVA was tested in both groups by intradermal skin tests. A 200 μg/mL OVA solution and saline (for control) were used. One hundred microliters of solutions (OVA and saline) were injected subcutaenously, symetrically in the shaved dermis on the back of each animals. The extent of the reaction was measured 1h later using a calliper. Two measurements were taken at right angles to one another, in order to calculate the wheal area (mm^2^) [13].

#### Bronchoalveolar lavage (BAL)

BAL was performed at the end of each acute experiment (from 1.5 to 2.5 hours after the last OVA challenge on day 28), following immediately the animal euthanasia. A polyethylene-190 catheter was advanced through the endotracheal cannula into the trachea and gently wedged into a bronchus. Slow injection (followed by removal aspiration) of 5 ml of HEPES solution (140 mM NaCl, 5 mM KCl, 1 MgCl2, 10mM glucose, and 10 mM HEPES; pH 7.4) was repeated three times (overall injected volume = 15ml). BAL was then collected into a tube placed on ice and previously filtrated on nylon tissue with 60 μm mesh to remove mucus. An aliquot of this suspension was then centrifuged (600 rpm, 10 minutes) and the resulting Cytospin cell preparations were stained with May-Grünwald-Giemsa.

#### Microscopy and cell counting protocol

Images were acquired by an Olympus CDD camera coupled to an Olympus microscope with a x40 objective, so that final pixel size in a 1360 x 1024 image was 0.32 μm. At least 100 cells were included in the differential cellular count: macrophages, lymphocytes, neutrophils, eosinophils, basophils, and monocytes were separately counted. Epithelial and unidentifiable cells were excluded.

### Anesthesia and surgical preparation before acute experiment

Anesthesia was induced with a mixture of intravenous (through the ear vein) pentobarbital (0.30 ml.kg^-1^) and intramuscular ketamine (0.10 ml.kg^-1^), and pursued with continuous pentobarbital infusion (0.6 ml.h^-1^). Anesthetic depth was quarterly evaluated (every 15 minutes) with the monitoring of physiological parameters (respiratory rate, heart rate) and the assessment of reflexes (palpebral reflex, corneal reflex, ear pinch withdrawal reflex and jaw tone). Supplementary anesthetic doses were administrated in case of too light anesthesia (intravenous pentobarbital bolus of 0.03 ml.kg^-1^ and intramuscular ketamine bolus of 0.01 ml.kg^-1^). At the end of the acute experiment, euthanasia was obtained by an overdose of Dolethal (bolus of 5 ml, Vetoquinol SA, Lure, France).

As previously described [8], the trachea was exposed via a midline incision, paying attention to preserve the integrity of vagal nerves. Rabbits were then tracheotomized and intubated with a steel tracheal cannula inserted caudally, allowing spontaneous breathing. Abdominal muscles electromyograms (EMG) was recorded by bipolar fine wire stainless steel electrodes (A-M Systems, INC, Carlsborg, WA 98324) introduced in either the transversus abdominis or external oblique abdominal muscles. Rectal temperature was continuously monitored with an electrical thermistor (Physiotemp Instruments, YSI 402 Clifton, NJ, USA) and maintained at 38°C using a heated operating pad, on which rabbits were laid in the supine position.

### Breathing pattern and respiratory resistance

The tracheal cannula was connected to a pneumotachograph (Fleisch # 0, Metabo, Hepalinges, Switzerland) to simultaneously record airflow and airway pressure. The flow signal was digitized at 200 Hz. Data was fed into a computer and integrated to volume. Tidal Volume (Vt) and flow were displayed continuously throughout the period of acquisition and were stored on disk for later analysis. Respiratory resistance (Rrs) was measured by an adjustment of the forced oscillation technique, as previously detailed [8, 14, 15], in order to evaluate the exercise induced bronchodilation.

### Rhythmic electrical stimulation of muscle contractions (EMC)

EMC was used as a valid model of exercise. We therefore used the validated approach of Cross et al. [16] adapted and formerly described in a previous study in rabbits [8]. Briefly, hindlimbs of anesthetized rabbits were shaved and fitted with stimulating surface electrodes (Dura-Stick Premium, REF 42205, DJO, USA) taped over the skin and connected to an electrical stimulator (Neuro Trac Rehab, Verity Medical LTD, UK). Stimulation allowing rhythmic hindlimb muscles contraction was maintained for 3 or 4 min bout, during which the current intensity was progressively increased from 10 to 30 mA, in order to maintain a sufficient increase in metabolism and corresponding hyperpnea (at least a 30% increase of resting minute-ventilation) [8, 16, 17].

### Tracheal stimulation and analysis of the cough reflex

The device designed to stimulate the trachea has been described in detail and validated in previous reports [8, 18–20]. A semi-rigid rotating silastic catheter introduced through the tracheotomy is driven by a small electrical motor (low voltage DC motor 719RE280, MFA/Comodrills, UK) that spins the catheter and rubs the catheter tip on the airway mucosa. The duration of probing randomly varied from 50 ms, corresponding to almost a single probe rotation, to 1000 ms.

Characterisation of a reference breath (at rest and during exercise) was obtained by averaging Vt and peak expiratory flow (V’epeak) on 3 consecutive respiratory cycles prior to tracheal stimulation [8]. Responses to tracheal stimulations were assessed by the change in Vt and V’epeak relative to the reference breath, as previously described [19, 21]. A significant response to tracheal stimulation was considered when the parameter (Vt, V’epeak) was situated outside the 99^th^ percentile (i.e. larger than the mean + 3 SD). Cough reflex (CR) was defined by a significant increase in both Vt and V’epeak and expiration reflex (ER) was defined by an isolated increase in V’epeak, not preceded by an increase in Vt. A “no response” (NR) pattern was defined by no change in tidal flow and volume. Cough threshold (CT) was defined as the shortest stimulus duration that induced a cough reflex response [21].

### Protocol

One data acquisition sequence consisted of a bout of rest followed by electrically induced muscle contractions (EMC) records (i.e. exercise) [8]. After an initial sequence allowing Rrs measurements, each rabbit underwent 2 to 3 sequences (separated by at least 10 min of recovery, i.e. without any stimulation) including tracheal stimulations. Various durations (50 ms, 150 ms, 300 ms, 600 ms, 1000 ms) of tracheal stimulations were randomly delivered at rest, while the animal had been breathing quietly for at least 1 min. All stimulations were applied either completely during inspiration (i.e. 50 ms to 300 ms) or almost starting during inspiration (i.e. 600 ms and 1000 ms). During EMC, 3 to 4 tracheal stimulations (from 50 ms to 1000 ms) were as well randomly delivered at intervals of 1 min.

### Data analysis

Statistical analysis was performed using the JMP 9.0.0 package (2010 SAS Institute Inc). Respiratory variables (Vt, minute ventilation V’e and Rrs) were averaged over the relevant study period: i.e. at rest and during exercise for comparisons between groups.

The incidence and type of response were analysed by χ^2^ test or the Fisher’s exact test. Analysis of variance (ANOVA) was used for the comparison of the breathing pattern (Vt, V’epeak) and respiratory resistance (Rrs). Comparisons of CT values between rest and exercise were performed using nonparametric Wilcoxon signed-rank test. The limit of significance was *p* < 0.05. Data are expressed as mean ± SD.

## Results

An overall of 494 tracheal stimulations were performed (271 at rest; 223 at exercise) and analyzed in 18 rabbits (10 OVA and 8 controls).

### OVA intradermal skin tests and BAL eosinophil count

OVA intradermal injection challenge was highly positive in OVA rabbits with a mean wheal area of 630 mm^2^ ± 297 mm^2^ and strictly negative in control rabbits (0 mm^2^, *p* < 0.0001). In addition, a significant increase of eosinophils (*p* = 0.008) occurred in BAL fluid in OVA rabbits compared to control ones (Table 1).

**Table 1:** Wheal surface of the intradermal skin tests, total BAL cell count and BAL eosinophil count of OVA and control rabbits, expressed as mean (Standard Deviation).

Condition	Wheal surface mm2 (SD)	Total BAL cell count (SD)/ml	BAL eosinophil count (SD) %
**OVA (n = 10)**	630 (297)	1.61 (57) x10^6^	26.3 (16)
**Control (n = 8)**	0 (0)	1.08 (43) x10^6^	2.5 (2.5)

BAL: Bronchoalveolar lavage

OVA: Ovalbumin-sensitized rabbits

### Breathing pattern and respiratory resistance at rest and during exercise (EMC)

**Fig 2:**
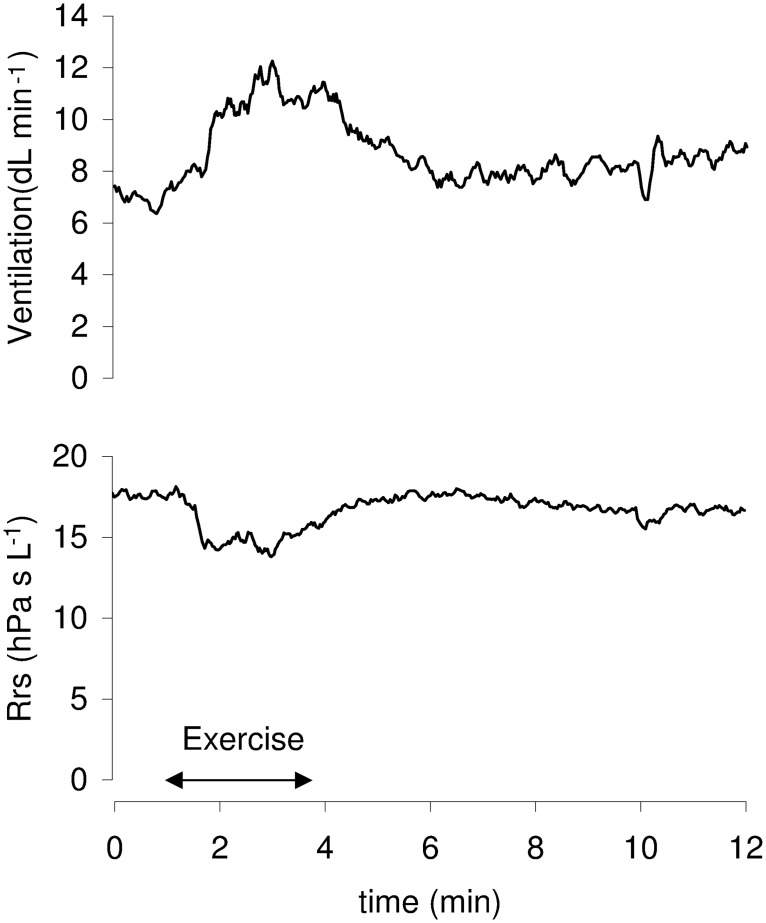
Sequence including rest, exercise and recovery. During electrically induced exercise (horizontal arrow), minute ventilation is increased and respiratory resistance (Rrs) is decreased, indicating concomitant bronchodilation.

Following rhythmic electrical stimulation of muscle contractions (i.e. exercise), the mean minute ventilation increased from rest, by 36% and 35% in OVA and control rabbits, respectively (Table 2). Concomitantly, the mean reduction in Rrs was 11% (OVA) and 13% (control) (Table 2; Fig 2). No significant statistical differences were found between groups.

**Table 2:** Minute ventilation and respiratory resistance at baseline and during exercise for OVA and control rabbits.

	Baseline	Exercise *(2&3min)*	p
	**Rrs (hPa s L**^**-1**^**)**		
**OVA (n = 10)**	16.8 ± 1.9	14.9 ± 2.1	0.0003
**Control (n = 8)**	17.9 ± 1.4	15.5 ± 1.3	< 0.0001
	**V’****e** **(mL min**^**-1**^**)**		
**OVA (n = 10)**	984 ± 232	1338 ± 229	< 0.0001
**Control (n = 8)**	794 ± 122	1075 ± 216	0.0008

OVA: Ovalbumin-sensitized rabbits

**V’****e**: minute ventilation

Rrs: respiratory resistance

### Types of ventilatory responses to tracheal stimulation

**Fig 3:**
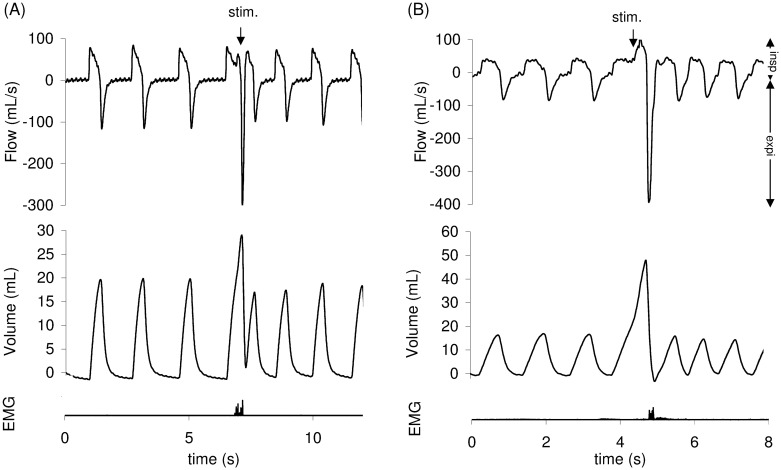
Cough reflex (CR) at rest (A) and during exercise (B). CR is characterized by an increase in both tidal volume (Vt) and peak expiratory flow (V’epeak). Abdominal muscles electromyogram (EMG) also showed activity on the stimulation breath. The downward arrow indicates the tracheal stimulation. Positive and negative airflow rates indicate inspiration and expiration, respectively.

Three types of response to tracheal stimulation were encountered: CR (Fig 3), ER and NR. Overall stimulations, as stimulations performed at rest and during exercise (EMC) in both (OVA and control) groups are presented in Table 3.

**Table 3:** Overall tracheal stimulations performed at rest and during exercise (electrically induced muscle contractions) for OVA and control rabbits (expressed as absolute value (percentage)).

	OVA (n = 10)	Control (n = 8)
**Total number of stimulations**	261 (53%)	233 (47%)
**Stimulations at rest**	141/261 (54%)	130/233 (56%)
**Stimulations during exercise**	120/261 (46%)	103/233 (44%)

OVA: Ovalbumin-sensitized rabbits

During exercise, in the OVA group, CT remained unchanged in 5 rabbits, decreased in 4 rabbits and increased in 1 rabbit, indicating no modification of the sensitivity of the cough reflex compared to baseline (no significant difference (NS)). For the control group, CT during exercise increased in 4 rabbits and remained unchanged in 4 rabbits, even so leading to a significant decreased sensitivity of the cough reflex compared to baseline (*p* = 0.0313, Table 4).

**Table 4:** Cough threshold (CT) at rest and during exercise for OVA and control rabbits. Data expressed in ms (mean ± SD).

	OVA (n = 10)	Control (n = 8)	p
**Rest-CT (ms)**	230 ± 294	62 ± 35	NS (0.1304)
**Exercise-CT (ms)**	190 ± 184	125 ± 46	NS (0.3463)

OVA: Ovalbumin-sensitized rabbits

CT: Cough threshold

## Discussion

To the best of our knowledge, our study is the first that focused on the influence of exercise on the cough reflex to tracheal mechanical stimulation in OVA-challenged rabbits. The study demonstrates that allergen-induced airway inflammation prevents the physiological exercise-induced decrease of the CR sensitivity, previously demonstrated [8].

Cough is a common symptom in a wide range of respiratory and non-respiratory diseases, but still remains a difficult clinical problem mostly because of the poor understanding of the underlying pathophysiological processes involved. Numerous animal models have been used to explore the mechanisms of cough but often using irritant compounds (such as citric acid or capsaicin) and rarely dealing with physiological triggers such as exercise is [22–27]. The inflammatory responses shown by the rabbit model, especially in the case of asthma, are comparable with those that occur in humans [28–30]. Indeed, neonatally immunized rabbits exhibit many of the features of human asthma, including airway hyperreponsiveness (AHR) to inhaled antigen [28–30], acute and late-phase airways obstruction [31] and pulmonary eosinophil and lymphocyte recruitment [28, 32], as well as the production of immunoglobulin E (Ig E) antibodies [31]. Since the first rabbit immunisation protocol [33], various antigens (including OVA) have been proposed to allow valuable animal experiment [34]. The OVA model of airway inflammation is characterised by high levels of OVA-specific IgE and eosinophils, a T-cell predominant bronchial inflammatory response, and the development of AHR, which are similar to characteristics of asthma in humans [26]. The rabbit model is also of particular interest because it allows spontaneous breathing (instead of mechanical ventilation for smaller animal models) during anaesthesia, leading to, repeated physiological measurements [34], as well as it shows a similar response to the drugs commonly used in asthmatics [34]. All these similarities between the allergic rabbit model and human asthma provide relevance to this model, therefore allowing the assessment of the modulation of cough during exercise in the present study.

Exercise represents a quantifiable and reproducible stressor that can be easily modified experimentally [35]. It is also able to trigger various and complex interacting mechanisms within the psycho-neuro-immune-endocrine pathways [36]. Exercise-induced bronchoconstriction (EIB) is common but key aspects of its pathogenesis are still lacking [37]. Regarding to the pathophysiological mechanisms involved, it is generally assumed that exercise-induced asthma/bronchoconstriction involves both osmolar (“airway drying”) and vascular (“thermal”) modifications of the airways, in addition to parasympathetic stimulation caused by the entrance of fresh air into the respiratory tract [38]. Both hypotheses are based on the marked hyper-ventilation during physical activity, leading to significant water and heat loss through respiration. Increased water loss is responsible for hyper-osmolality of the extracellular fluid lining the bronchial mucosa [39] further leading to the release of inflammatory mediators from mast cells, eosinophils, neutrophils, and other inflammatory cells, including newly formed eicosanoids [40, 41]. The epithelium may act as a key regulator of the balance of eicosanoids in the airways by both the induction of the release of constrictive eicosanoids and by the downregulation of the release of the relaxant PGE_2_ [42]. Several airway TRP ion channels have been shown to sense and react to ambient air temperature opening new windows in the understanding of the pathogenesis in a diversity of airway reactions, particularly cough, appearing in many common respiratory diseases [43].

Only poor information is available regarding to the modulation of cough during human activities [5] but common low-level stimuli such as eating, drinking, talking, laughing, singing or exercising have been shown to cause cough, suggesting the emergence of a new concept (even if little explanation has so far been suggested) where cough is considered as a neuropathic disorder [44]. In the field of exercise (although available information is limited and often contradictory), present knowledge seems to indicate that the various adaptive responses brought into action by physical activity may modulate the CR [5], supporting that cough reflex exhibits plasticity at peripheral and central levels [45]. In healthy subjects, it has been shown that cough threshold to distilled water aerosol was increased during exercise and voluntary isocapnic hyperpnea [7]. Our results confirm a physiological decrease in sensitivity (i.e. increased in cough reflex threshold) of the CR during exercise (compared to sensitivity at rest) in anesthetized rabbits, as previously described [8]. Overall, it appears that cough is depressed (i.e. down-regulated) when ventilation increases, at least during isocapnic hyperpnea triggered by exercise.

Exercise-induced cough is frequent in asthmatic patients [9] or even in athletes developing airway inflammation [10], suggesting the potential of exercise to trigger cough. Our study shows an unchanged CR sensitivity during exercise in the OVA sensitized and challenged rabbits in contrast with the decrease of CR in the control animals. The impact of exercise in the modulation of cough is often discussed with respect to the state of contraction/relaxation of airway smooth muscle as a consequence of the numerous adaptive responses (involving neural and mechanical mechanisms) generated during physical activity and principally leading to exercise-induced bronchodilation [14,46,47]. As bronchoconstriction is able to modulate cough by enhancing the CR [48], one might expect that bronchodilation would decrease cough induced by mechanical stimulation of the airways [8]. Indeed, both groups (OVA and Control) showed a similar bronchodilation during exercise as suggested by the significant decrease of respiratory resistance (Table 2). There was also no difference either in the increase of V’e or the decrease of Rrs between OVA and control rabbits. With regard to this comparable state of hyperventilation and bronchodilation during exercise, the modulation of cough appeared however different between OVA (lack of desensitization) and controls (desensitization). Bronchodilation *per se* unlikely explains the differences in the effect of exercise on the CR. Some human studies have pointed out that bronchodilation may fail to affect CR sensitivity [49], as well as exercise without evidence of bronchodilation may increase cough threshold [7].

Our results highlight the contribution of allergen-induced experimental airway inflammation in the modulation of the CR during exercise. It has been shown that cough is associated with chronic eosinophilic disorders (such as asthma, cough-variant asthma or even eosinophilic bronchitis) [30, 31]. However, the relationship between cough receptor sensitivity and eosinophilic inflammation of the airway in patients with asthma or allergic rhinitis still remains unclear [50]. It is hypothesized that eosinophils in contact with sensory airway nerves [51] cause an eosinophil-dependent neuro-inflammation able to enhance cough [52]. Even the specific mechanisms by which this inflammation modulate the CR have not been established, it however seems that inflammatory mediators, indirectly released, sensitize airway neuronal responses to capsaicin involving the activation of the transient receptor potential cation channel subfamily V member 1 (TRPV1) [44, 53]. In a previous study in Brown-Norway rats [37], no control rats with or without exercise showed evidence of Exercise-Induced Bronchoconstriction (EIB) but 80% of the OVA-sensitized rats demonstrated abnormal breath sounds upon exposure to aerosolized OVA and 30% of OVA-sensitized rats exposed only to exercise had abnormal breath sounds. Lung tissue levels of TNF-α, IL-1α, growth-related oncogene/keratinocyte/chemoattractant, and IFN-γ were significantly higher (P < 0.001) in the OVA-sensitized group. Most of these cytokines were not altered in the OVA-sensitized rats exposed only to exercise, suggesting a different mechanism of EIB [37].

If the evidence of an airway inflammation in OVA rabbits suggests a modulation of cough at a peripheral sensory nerves level, the contribution of a modulation at a central level should also be questioned. Indeed, the brainstem respiratory network should be considered as a key integrator regarding to the different afferent inputs upstream from triggering defensive airway reflexes. Moreover, coughing and breathing are initiated by neurons situated in very close (even overlapping) areas of the brainstem [54, 55]. Thus, facing a tracheal mechanical stimulation, the central respiratory integrator should trigger the most adapted motor response proper to the prevailing needs of both conditions (i.e exercise and rest) [8, 55]. The decrease of cough during exercise in control rabbits then suggests that the respiratory pattern generator favours an increase of respiratory activity and inhibits airway defensive reflexes, whereas in OVA rabbits the airway inflammation prevents the development of the exercise-driven inhibition of the defensive reflexes. This lack of desensitization of cough during exercise in OVA rabbits would make teleological sense because airways remodelling associated with airway inflammation may be considered as a weak point (facing the potential insult to the airways caused by hyperventilation during exercise [56]), allowing the preservation of defensive reflexes.

## Conclusions

Our results confirm a decrease of cough during exercise in control rabbits and suggest for the first time the lack of desensitization in OVA-sensitized and challenged rabbits. This result contributes to explain the higher susceptibility to cough of asthmatics during exercise. Further studies are needed to explore the underlying inflammatory mechanisms contributing to the absence of the CR desensitization physiologically-associated to exercise.
